# Consumption of medical resources and outcome of shoulder disorders in primary health care consulters

**DOI:** 10.1186/1471-2474-14-348

**Published:** 2013-12-13

**Authors:** Juha Paloneva, Sanna Koskela, Hannu Kautiainen, Mauno Vanhala, Ilkka Kiviranta

**Affiliations:** 1 Department of Surgery, Unit of Family Practice, Central Finland Central Hospital, Keskussairaalantie 19, Jyväskylä 40620, Finland; 2 Division of Orthopaedic and Trauma Surgery, Department of Surgery, Oulu University Hospital, Oulu, Finland; 3 Unit of Primary Health Care, Kuopio University Hospital, Kuopio, Finland; 4 Unit of Family Practice, Central Finland Central Hospital, Keskussairaalantie 19, Jyväskylä 40620, Finland; 5 Department of Orthopaedics and Traumatology, University of Helsinki and Helsinki University Hospital, Helsinki, Finland

**Keywords:** Shoulder, Rheumatology, Primary health care, General practice, Prospective cohort study, Outcome, ASES, VAS

## Abstract

**Background:**

Shoulder disorders are common problems in primary health care. The course of disease of patients consulting for a new episode of a shoulder problem has been thought to be benign. In this prospective cohort study, we assessed the one-year consumption of medical resources and clinical outcome of shoulder disorders inclusive of all disease episodes.

**Methods:**

All individuals consulting primary health care for shoulder disorder in a catchment area of more than 120 000 people were included. A composite questionnaire including the American Shoulder and Elbow Surgeons Standardized Shoulder Assessment Form (ASES) was used to measure use of resources as well as shoulder pain and function. A follow-up assessment was performed after one year.

**Results:**

A total of 128 individuals responded to the questionnaire. Only 24% of the patients had recovered after one year. Mean shoulder pain (Visual analogue scale, VAS, max 100 mm) decreased from 38.9 mm to 28.6 mm (95% CI -16.3 to -4.2 mm). The ASES score (max 100) improved significantly from 59.9 to 70.2 (95% CI 5.3 to 15.3). Mean one-year consumption of medical resources after the index consultation was 1.5 consultations, 0.5 radiological examinations, and 3.3 visits to physiotherapist. Mean resource-weighted direct costs were €543/patient/year (95% CI €351 to 735).

**Conclusions:**

Shoulder disorders are often chronic and require a significant amount of resources from the health care system. The clinical outcome of the management of shoulder disorders in our study population including also individuals who have consulted previously for a shoulder problem is notably poorer than the one reported by previous studies on new episodes. However, despite the relatively modest outcome, subjective disability is low.

## Background

Shoulder disorders related to pain and limited range of motion are common complaints in the general population as well as in primary health care. Epidemiological data show prevalences of 7% for both pain and limited range of motion [1,2]. The proportion of individuals consulting primary health care for shoulder problems is substantially lower than the population-based prevalence of shoulder pain or mobility restriction. The annual prevalence and incidence of people consulting for a shoulder condition has been reported to be 2.4-4.8% and 1.1-2.9%, respectively [3-5]. Shoulder disorders constitute a significant public health problem, as they are among the most common musculoskeletal disorders.

The objective of the management of shoulder problems is to control pain and restore or maintain function of the shoulder joint. A multidisciplinary approach is generally recommended in the treatment of these mostly degenerative conditions. The first lines of treatment are relative rest, pain medication (non-steroidal anti-inflammatory drugs or paracetamol), local corticosteroid injections, and physiotherapy. The majority of patients can be managed using this approach. A specialist’s evaluation may be necessary for those with persistent manifestations despite adequately performed conservative treatment [6]. Surgical procedures may be considered when conservative management fails.

The course of shoulder disorders in general practice has generally been thought to be rather benign [7,8]. Half of the individuals with a new episode of a shoulder disorder recover completely within 6 months and almost half consult only once [9,10]. However, the previous literature suggests that for a significant proportion of individuals their shoulder problems may be continuous and long-term [9]. The outcomes of the management of shoulder disorders have been reported heterogeneously and comparable data are scarce.

The current literature is limited to the prognosis of new episodes of shoulder disorders in primary health care [5,7,8,11-14]. Due to the chronic nature of shoulder disorders, both resource use and clinical outcome are potentially underestimated if only new episodes are observed. The need for medical care has not been reported in a patient population that has included both new episodes and individuals who consult continuously for their disease. Our research question was to determine if the consumption of medical resources, direct costs and clinical outcome of shoulder disorders in primary health care consulters during a one-year follow-up, inclusive of all disease episodes, is different compared with previous studies on new disease episodes.

## Methods

### Design

The study population of this prospective cohort study in the primary health care setting comprised two community-based health centres, located in Jyväskylä, Finland, serving a total population of approximately 120 000 individuals. In Finland, occupational medicine clinics (classified as primary care) are alternatives to public health centres for a large proportion of the working age population. Therefore, two occupational medicine clinics serving a total population of 15 000 individuals were included. The populations of these health centres and occupational health clinics partially overlap. A prospective cohort study was selected to obtain detailed information on the use of medical resources and clinical outcome of shoulder disorders in our study population.

The term “shoulder disorder” was defined as pain in the deltoid and upper arm region, recurrent instability of the glenohumeral or acromioclavicular joint, or weakness or restricted mobility of the shoulder. Acute fractures and dislocations were excluded.

### Data collection and outcome measures

All patients consulting a general practitioner or specialist in occupational medicine for a shoulder disorder during six months between October 2007 and March 2008 were invited to participate. During their enrolment in physician’s practice (index visit), all patients consulting for shoulder disorders received a self-administered questionnaire. After the index consultation, the patients completed the composite questionnaire (baseline assessment) consisting of demographic information at home and returned it. The first part of the questionnaire concerned demographics including name and personal identification number, education (years), smoking status (yes/no) and employment status (working, unemployed, sick leave, student, pensioner). The second part contained specific items on the shoulder disorder: side of the affected shoulder (right/left) and information about the dominant hand (right/left); duration (months/weeks) and frequency (less than once per month, repeatedly, continuously) of the disorder and an estimation of its cause (no reason, physical strain, injury, other); the influence of the shoulder problem on the ability to perform normal activities (unable, significant difficulties, slight difficulties, no difficulties); history of glenohumeral joint dislocations (yes/no); surgical procedures on the same shoulder (type and place of operation); radiological examinations performed (plain radiograph, ultrasonography, CT, MRI); number of visits to physiotherapist, frequency of home-based exercises (times per week); consultations due to problems in the same shoulder during the previous 12 months (number); referral to specialised care (yes/no); number of days on shoulder-related sick leave during the previous 12 months.

After one year, a follow-up questionnaire, covering the same items as the baseline questionnaire, was sent to all the individuals who had returned the baseline assessment form. A follow-up period of one year was selected to determine the clinical outcome as well as the annual costs and consumption of medical care. In addition, the patients’ perception of recovery was assessed (yes/no). The data were stored in a structured, closed database for statistical analyses.

The patient self-report section of the American Shoulder and Elbow Surgeons Standardized Shoulder Assessment Form (ASES, 0–100 points, 100 = best result) was utilized as an outcome tool to measure functional limitations and pain in the shoulder. The subjective patient self-report section consists of two equally weighted domains, pain and function. It is widely used for clinical outcomes assessment in patients with shoulder instability, rotator cuff disease, and glenohumeral arthritis [15,16]. Pain was evaluated using the Visual Analogue Scale (VAS, 0-100 mm, 0 = no pain, 100 = worst possible pain).

The main determinants in our study are the change in pain and ASES during one year follow-up. Confounding factors include inability to contact all subjects with a shoulder disorder and a potential tendency to not answering the follow-up questionnaire if the symptoms have been relieved.

### Costs

Direct costs per patient were determined by calculating the sum of costs (year 2011) produced by different treatment (primary care consultations, specialist’s consultations, visits to physiotherapist, operative treatment) and examination modalities (radiological examinations). Absorption costs were used to determine the costs of a visit to GP (€102), specialist in physical and rehabilitation medicine or orthopaedic surgeon (€289), and physiotherapist (€45) as well as plain radiography (€52), ultrasonography (€82), CT (€116), and MRI arthrography (€427). Diagnosis Related Groups (DRGs) were used to determine the direct costs of operative treatment. DRGs are classification systems that group patients according to the resources required for their treatment and their clinical characteristics (principal diagnosis, ICD diagnoses, gender, age, treatment procedure, discharge status, and the presence of complications or comorbidities) (http://www.nordcase.org) [17]. Diagnoses and procedure codes regarding the surgical operations were verified from the patient’s health records. The costs were obtained using VisualDRG software (Datawell Ltd, Espoo, Finland) based on DRGs, cost weights and prices.

### Statistical analyses

Two main determinants were used in the statistical analyses. 1) The research subjects were divided in two subgroups based on the number of consultations (no consultations, one or more consultations during one year) before the index visit. 2) As duration of symptoms before the first consultation has been thought to be one of the most important prognostic determinants, the research subjects were divided in two subgroups according to symptom duration (equal to or less than 6 months, over six months) before the index visit.

Comparisons between groups in the categorical outcomes were performed by the chi-square test or the Fisher-Freeman-Halton test. For the continuous outcomes, the t-test, analysis of variance or covariance was used. In case of violation of assumptions, the permutation test, the Wilcoxon rank-sum test or the Kruskal-Wallis rank test was used instead. Changes within groups were tested by the paired samples t-test or the McNemar test. The confidence intervals for costs were obtained by bias-corrected bootstrapping (2000 replications) and the difference between the groups in costs by the bootstrap type t-test. Incompletely reported data items were left out of the analyses. P-value less than 0.05 was considered statistically significant. Stata 11.1 was used for statistical analyses (StataCorp, TX, USA).

### Ethical aspects

The study was conducted according to the Declaration of Helsinki. Participation was voluntary. A written informed consent was obtained from all individuals participating in the study. Ethical approval was given by the Regional Ethical Review Board of Central Finland Health Care District, Jyväskylä, Finland, Dnro 23/2007.

## Results

After the index visit, 128 patients returned the baseline questionnaire. The return percentage from the health centres was 78. The follow-up questionnaire one year later was returned by 104 individuals (81%) after two reminders. Background information about the study population is presented in Table 1.

**Table 1:** Data on the patients at index visit (n = 128)

**Variable**	**Value**
Number of female, (%)	86 (67)
Age, mean (SD)	54 (15)
Smokers, %	25 (20)
Employment status*,* n (%)	
Working	59 (46)
On sick leave	4 (3)
Unemployed	10 (8)
Pension (full or part time)	47 (38)
Students	5 (4)
Education years, median (IQR)	12 (9–15)
Dominant side affected, n (%)	84 (66)
Duration of symptoms, mo, median (IQR)	6.0 (2.0-12.0)
Equal to or less than 3 mo, n (%)	43 (34)
Over 3 but less than 6 mo, n (%)	26 (20)
Over 6 mo, n (%)	59 (46)
Surgical procedure performed, n (%)	12 (9)
Cause of symptoms, n (%)*	
Not apparent	63 (49)
Injury	32 (25)
Physical strain	37 (30)
Glenohumeral joint dislocation	13 (10)

*More than one could be named.

IQR, inter quartile range.

### Clinical outcome

Pain (VAS) decreased significantly during the one-year follow-up period from 38.1 (SD 24.6) to 28.6 mm (SD 29.1) (95% CI -15.1 to -3.0, p = 0.0041) across the study population. During the follow-up period, the mean ASES score improved significantly from 59.9 (SD 19.3) to 70.2 (SD 22.9) (95% CI 5.3 to 15.3, p < 0.001). The subgroup analyses are presented in Table 2.

**Table 2:** Change in VAS and ASES after one year according to (a) number of consultations and b) duration of symptoms before index visit

**a)**	**Index, mean (SD)**		**Follow-up, mean (SD)**		**Change, mean (95% CI)**		p*
**Group**	**A**	**B**	**A**	**B**	**A**	**B**	
ASES	66.5 (17.3)	56.7 (19.6)	72.7 (19.4)	69.0 (24.6)	6.2 (-1.0 to 13.4)	12.3 (5.6 to 18.9)	0.26
VAS, mm	33.9 (25.6)	40.3 (25.1)	28.8 (24.2)	29.3 (28.3)	-5.1 (-14.4 to 4.3)	-11.0 (-18.9 to -3.0)	0.37
**b)**	**Index, mean (SD)**		**Follow-up, mean (SD)**		**Change, mean (95% CI)**		p*
**Group**	**C**	**D**	**C**	**D**	**C**	**D**	
ASES	60.6 (20.2)	59.0 (18.8)	74.6 (21.1)	67.5 (22.8)	14.0 (5.0 to 23.0)	8.5 (2.9 to 14.4)	0.28
VAS, mm	38.5 (25.9)	39.2 (23.8)	23.1 (22.3)	33.4 (29.0)	-15.4 (-26.2 to -4.6)	-5.8 (-12.4 to 0.7)	0.12

a) Not consulted before the index visit (group A), one or more consultations before the index visit (group B); n (ASES) = 27 (A) and 56 (B); n (VAS) = 28 (A) and 57 (B).

b) Duration of symptoms ≤6 months (group C) and > 6 months (group D) before index visit; n (ASES) = 37 (C) and 43 (Group D); n (VAS) = 38 (C) and 44 (D).

*Between groups.

A total of 24% of the patients considered themselves completely recovered during the one-year follow-up period. Initially, 84% of the patients reported frequent or continuous complaints. After one year, the corresponding proportion had fallen to 68% (p < 0.001). In the baseline questionnaire, 67% of the individuals had reported no problems or only slight difficulties in normal activities. After one year, the proportion had risen to 81% (p = 0.017).

Subgroup analyses showed that 22% of the individuals with a new episode of shoulder pain and 25% of the patients who had been consulting before the index visit completely recovered during the follow-up period (p = 0.77 between groups). A total of 29% of the individuals with a duration of symptoms ≤ 6 months before the index visit, and 20% of those who had been suffering from shoulder complaints for over 6 months recovered completely during the follow-up period (p = 0.31 between groups).

Before the index visit, 25% of all the employed individuals had been on sick leave due to shoulder problems for a median of 21 days (IQR 7–90) during the past 12 months. During the follow-up period, 18% required sick leave due to shoulder disorders. The median duration of sick leave was 70 days (IQR 11–220).

The baseline assessment did not show statistically significant differences in the demographical data, pain and ASES score between the individuals who responded to the follow-up questionnaire and those who did not.

### Consumption of medical resources

In total, 62% of the patients had consulted a physician during the previous 12 months due to problems in the same shoulder before the index visit. Half of the patients (51%) consulted a physician during the follow-up period after the index visit (median twice, IQR 1–3, mean 1.5). Twenty percent of the patients were referred to a specialist in physical and rehabilitation medicine or an orthopedic surgeon. A surgical procedure of the shoulder under anesthesia was performed on 9% of the patients before the index visit and on 8% during the follow-up period. Radiological imaging was performed on 39% of the individuals during the follow-up period. Plain radiography accounted for 46%, ultrasonography 35%, MRI 15%, and CT 4% of all radiological examinations. Initially, 17% of the individuals had performed exercise therapy. During follow-up, 47% participated in exercise therapy for a median of five times (IQR 2–10). The average one-year consumption of medical resources per patient after the index visit was 1.5 consultations, 0.5 radiological examinations, and 3.3 visits to a physiotherapist (Table 3).

**Table 3:** Shoulder-related consumption of medical care/patient/year after first consultation according to number of consultations and duration of symptoms before baseline

	Previous consultations*		**p**	**Duration of symptoms**		**p**
	**No (n = 36)**	**Yes (n = 68)**		**≤6 months (n = 48)**	**>6 months (n = 52)**	
Physician consultations, mean (range)	1.1 (0–9)	1.8 (0–15)	0.21	1.3 (0–9)	1.7 (0–15)	0.51
Referral to specialist, n (%)	6 (17)	14 (22)	0.60	7 (15)	11 (22)	0.37
Surgical procedures, n (%)	1 (3)	7 (10)	0.26	3 (7)	5 (10)	0.72
Visits to physiotherapist, mean (range)	2.8 (0–28)	3.5 (0–37)	0.67	3.2 (0–28)	3.3 (0–37)	0.97
Radiological examinations, n (%)						
Plain radiograph	10 (29)	12 (20)	0.28	11 (25)	9 (18)	0.44
Ultrasonography	3 (9)	14 (23)	0.10	9 (20)	7 (14)	0.43
CT	0 (0)	2 (3)	0.54	0 (0)	2 (4)	0.50
MRI	3 (9)	4 (7)	0.70	4 (9)	3(6)	0.70

*During 12 months before the index consultation.

### Costs

The average one-year direct costs associated with the treatment were €543 /patient/year (95% CI €351 to 735) across the study population. The costs for individuals with no previous consultations were €387 (95% CI €197 to 651) and €625 (95% CI €410 to 937) for those with consultations before the index visit (p = 0.19 between groups). The one-year costs were significantly lower in recovered (€242, 95% CI €103 to 478) than not recovered individuals after one year (€640, 95% CI €425 to 902), with a mean difference between groups of €398 (95% CI €91 to 714, p = 0.030). A third (37%) of the individuals in our study population did not incur direct costs after the index visit. A total of 15% of the patients generated 69% of the total costs (> €1000/patient) during the one year following the first consultation. The most important cause of high costs was a surgical procedure.

## Discussion

In the present study, we characterized the consumption of health care resources and outcome of shoulder disorders in primary care consulters during a one-year follow-up. Contrary to the previous studies, which have focused solely on new episodes, we also included individuals who had previously consulted a physician for their shoulder condition. New episodes accounted for a minority of the population consulting primary care in our study. We also found that the clinical outcome in our research subjects was substantially poorer than outcomes reported in previous studies, which included only new episodes of shoulder complaints.

### Consumption of medical resources

Our data show that shoulder disorders require a significant amount of multidisciplinary resources from the health care system. The one-year mean number of consultations and visits to a physiotherapist in our research material were on the same level as reported during six months in previous studies [10,18]. The rate of referrals to an orthopaedic surgeon or specialist in rehabilitation medicine in our material was higher (20%) than reported in the literature on new episodes of shoulder problems (5-14%). Surgery was also required more often (8% versus 0.38-2% in the literature) [3,7,10,19].

### Costs

The one-year direct costs in our study were relatively low but somewhat higher (€543) than those generated over six months in two previous studies on new episodes of shoulder problems (€250-326) [10,18]. The total costs associated with shoulder disorders in our material are likely to be significantly higher. In a previous study on new episodes, indirect costs constituted 84% of the total costs incurred as a result of shoulder-associated sick leave [10].

### Clinical outcome

The previous literature has focused on the prognosis of a new episode of shoulder pain in primary care with follow-ups of 6 months to a few years. Long duration of symptoms, high pain intensity and disability score, and middle-age have been associated with an unfavorable long-term outcome at initial presentation [9]. According to the previous literature, over half of the patients completely recovered over a period of 6–18 months [3,8,13,14]. This suggests a rather benign course of disease. However, shoulder disorders were chronic in the majority of our cases. Only 24% of our patients completely recovered. These observations suggest a substantially poorer outcome. The difference is not explained by the presence of individuals who had consulted a physician before the baseline visit, since the outcome according to pain and function improved significantly only in individuals who did not have a new episode (Table 2). Shoulder disorders did not appear to cause significant disability in our study population, as only 19% reported severe problems during their normal activities despite the presence of repeated or continuous symptoms in the majority (68%) at the one-year follow-up.

Differences in the treatments and clinical outcome in our study potentially reflect the different background of our research subjects compared with those in other studies. Previous studies concentrated on new episodes of shoulder disorders whereas we included all episodes regardless of the number of consultations before the index visit. Chronic symptoms (duration of symptoms three months or over, 66%) were more common in our sample than in previous reports (15-25%) [11,13,14]. The long duration and severity of the symptoms in the present patients may have lead to more active treatment than mild manifestations, which potentially would explain the higher percentage of referrals to specialised care and surgical procedures in our study compared with the previous literature.

The ASES value remained substantially below normal (92.2-93.4) even after the one-year follow-up irrespective of the duration of symptoms or number of consultations before the baseline [20]. Interestingly, only the patient group that had consulted a physician before the index visit showed a significant improvement in pain and function. According to different sources, the minimal clinically important change in the ASES scale is 6.4 or 12–17 points [16,21]. The clinical significance of the improvement in the ASES score thus remains borderline in our study. The improvement in disability indicates that the commonly implemented interventions for shoulder disorders have a positive, but limited, effect.

### Sick leave

The median duration of sick leave in our material (2.3 months) is remarkably longer than reported in the literature (a few days in most cases) [22]. This may reflect the different background (more chronic symptoms) of our research subjects or differences in social and health care systems between Finland and the countries of previous research populations.

### Strengths and limitations

A strength of our study is the use of the ASES outcome measure tool. The ASES has been evaluated in outpatient clinics patients upon referral to physical therapy for both operative and nonoperative diagnoses as well as in patients undergoing shoulder surgery. The ASES has been shown to be a reliable, valid and responsive tool in patients with rotator cuff disease, shoulder instability, glenohumeral arthritis, adhesive capsulitis, post surgical and other conditions [15,16]. However, ASES has not been validated in our particular research setting and population. Another advantage is that the patients filled in the questionnaire at home. Therefore, the answers are not likely to be influenced by the patient’s wishes regarding a potentially desired treatment or examination, or social benefits.

One potential source of bias is that our study material does not represent the whole population consulting for shoulder disorders because we were unable to contact all patients. This resulted in a relatively small sample size. The number of participants is not representative of the prevalence of the disorder in the population of the health center and occupational centers.

Another limitation is that some patients did not return the follow-up questionnaire. The course of disease in these individuals cannot be estimated. However, individuals who have experienced a significant relief of symptoms may refuse to return the follow-up questionnaire more often than those who still suffer from remarkable shoulder problems. The occupational medicine clinics were unable to provide a reliable number of individuals who had been approached regarding the baseline questionnaire. However, the majority of responses (78%) were obtained from the health centres, suggesting that the occupational medicine clinics were of a minor significance regarding this issue.

We did not aim at a specific diagnosis for our patients. It has been reported that general practitioners seldom make a specific diagnosis for shoulder disorder, and that even a general diagnostic category has been shown to change over time in a significant proportion of individuals [7,13,23,24]. Diagnosis based on clinical examination performed even by an especially trained physician is frequently unreliable [13,23]. Due to the lack of specific diagnoses it is difficult to determine whether these patients were managed according to the recommendations. Our research setting does not allow us to determine whether the relatively modest outcome reflects ineffective treatment methods or defective compliance of patients with the recommended therapy.

A shortcoming of the ASES scale, like most other shoulder outcome measure tools, is the lack of a validated definition of the functional capacity indicated by a certain ASES index.

## Conclusions

Shoulder disorders are frequently encountered problems in primary health care. New episodes in the present study represented a minority of the individuals consulting for a shoulder condition, as the majority of shoulder patients consulted more than once. Shoulder disorders did not cause significant disability despite the relatively poor prognosis, presence of chronic symptoms and reduced level of function in the majority of the patients after a one-year follow-up. Shoulder patients in our study required a significant amount of medical resources. However, mean annual costs were relatively low.

## Competing interests

The authors declare that they have no competing interests.

## Authors’ contributions

JP carried out the data collection, analysis and participated in manuscript writing. SK participated in data analysis and manuscript writing. HK participated in study design and performed statistical data analysis. MV and IK participated in study design and coordination and helped in drafting the manuscript. All authors read and approved the final manuscript.

## Pre-publication history

The pre-publication history for this paper can be accessed here:

http://www.biomedcentral.com/1471-2474/14/348/prepub
